
JOURNAL INFORMATION
==============================
NLM Title Abbreviation: Wirtschaftsdienst
Journal ID: Wirtschaftsdienst
NLM Journal ID: 101086612

Wirtschaftsdienst (Hamburg, Germany : 1949)

ISSN: 0043-6275

ARTICLE INFORMATION
==============================
PMCID: PMC7237610
PMID: 32454544
DOI: 10.1007/s10273-020-2654-y
Article ID: 2654
Article version: 1
Subjects: Zeitgespräch

Welthandel post Coronam

Felbermayr Gabriel gabriel.felbermayr@ifw-kiel.de

grid.462465.7 0000 0004 0493 2817 Institut für Weltwirtschaft, Kiellinie 66, 24105 Kiel, Deutschland
Electronic publication date: 2020 May 20
Print publication date: 2020
Volume: 100
Issue: 5
First page: 340
Last page: 343
Copyright: © Der/die Autor(en) 2020
License: Open Access Dieser Artikel wird unter der Creative Commons Namensnennung 4.0 International Lizenz veröffentlicht, welche die Nutzung, Vervielfältigung, Bearbeitung, Verbreitung und Wiedergabe in jeglichem Medium und Format erlaubt, sofern Sie den/die ursprünglichen Autor(en) und die Quelle ordnungsgemäß nennen, einen Link zur Creative Commons Lizenz beifügen und angeben, ob Änderungen vorgenommen wurden.
Die in diesem Artikel enthaltenen Bilder und sonstiges Drittmaterial unterliegen ebenfalls der genannten Creative Commons Lizenz, sofern sich aus der Abbildungslegende nichts anderes ergibt. Sofern das betreffende Material nicht unter der genannten Creative Commons Lizenz steht und die betreffende Handlung nicht nach gesetzlichen Vorschriften erlaubt ist, ist für die oben aufgeführten Weiterverwendungen des Materials die Einwilligung des jeweiligen Rechteinhabers einzuholen.
Weitere Details zur Lizenz entnehmen Sie bitte der Lizenzinformation auf http://creativecommons.org/licenses/by/4.0/deed.de.
License URL: https://creativecommons.org/licenses/by/4.0/

issue-copyright-statement: © The Author(s) 2020

==============================

Eigentlich sollte das Jahr 2020 ein Jahr des Feierns für die Welthandelsorganisation (WTO) werden. Vor 25 Jahren nahm sie ihre Arbeit auf, nachdem sie während der Uruguay-Runde in zähen, acht Jahre währenden Verhandlungen, gegründet wurde. Zum Feiern gibt es leider wenig Anlass – vor dem Ausbruch der Corona-Krise dämpfte der militante handelspolitische Unilateralismus der USA, insbesondere die Blockade der Berufungsinstanzen, und die damit verbundenen Kollateralschäden die Feierlaune; jetzt, inmitten der größten Rezession der Nachkriegsgeschichte setzt unkoordinierte, nationalstaatlich organisierte Gesundheitspolitik die Prinzipien der multilateralen Handelsordnung weiter unter Druck.

Die Malaise kündigte sich schon lange vor der Corona-Krise an. Die Globalisierung – charakterisiert durch ein schnelleres Wachstum internationaler Transaktionen relativ zu heimischen – ist schon seit 2010 in einer Phase der Verlangsamung, in der Ära der „Slowbalisation“, wie das britische Wochenblatt „The Economist“ im Januar 2019 formulierte. Gerade der Güterhandel, für den die WTO besonders ausgereifte und erprobte Regeln aufzuweisen hat, trägt zur neuen Stagnation bei; der deutlich weniger liberalisierte und international geregelte Dienstleistungshandel erweist sich indes als erheblich dynamischer, wohl angetrieben von neuen technologischen Entwicklungen. Die Ära Trump hat, jedenfalls in ihren ersten drei Jahren, noch keinen weiteren Trendbruch gebracht; jedenfalls nicht, wenn man auf globale Daten blickt.

Vergleicht man das Wachstum eines preisbereinigten Index der globalen Güterimporte mit dem eines Mengenindex des industriellen Outputs, so zeigt sich, dass der Handel spätestens seit 2010 nicht mehr schneller wächst als die Produktion; in der Zeit der Hyperglobalisierung (1990 bis 2008) überstieg die jährliche Wachstumsrate des Welthandels jene der Produktion im Durchschnitt um 3 Prozentpunkte.

Die Verlangsamung hat verschiedene Gründe; manche sind wohlfahrtsökonomisch unproblematisch, andere jedoch höchst bedenklich. In vielen Schwellenländern, allen voran in Ostasien und hier insbesondere in China, sind seit 2010 die Löhne deutlich stärker gestiegen als in den Staaten der Organisation für wirtschaftliche Zusammenarbeit und Entwicklung (OECD), denn die Ausstattungen mit Human- und Sachkapital relativ zu Arbeit sowie die technologischen Möglichkeiten vieler wichtiger Volkswirtschaften haben sich angeglichen. Die Handelstheorie sagt unter diesen Bedingungen voraus, dass der auf der Ausnutzung komparativer Vorteile beruhende Handel zurückgehen, und der auf Produktdifferenzierung sowie Skalenvorteilen beruhende Handel zunehmen sollte. Daher ist es nicht verwunderlich, dass der Nettoeffekt dieser beiden Kräfte eine Verringerung des Wachstumstempos des Handels zur Folge hatte.

## Rückkehr protektionistischer Politik

Diese Veränderungen sind im Kern zu begrüßen, auch wenn sie in vielen alten Industriestaaten zu unguten Verteilungseffekten führten, weil Arbeitnehmer in den vormals florierenden Exportsektoren Reallohneinbußen erlitten. Daneben ist aber seit 2008 eine zuerst schleichende und in den jüngeren Jahren eine sehr explizite Rückkehr protektionistischer Politik zu beobachten. Während in den Jahren nach 2008 in vielen Ländern industriepolitische Bestimmungen wie jene des „Buy American Act“ Präsident Obamas sowie Handelsschutzinstrumente wie Antidumpingzölle vermehrt zum Einsatz kamen, steigt seit dem Amtsantritt von Präsident Trump der weltweite Durchschnittszoll wieder an. Dazu kommt, dass es seit Gründung der WTO nicht gelungen ist, die Regeln des Welthandels zu reformieren, und den sich verändernden Umständen anzupassen. Das ging eine Weile gut. Doch spätestens seit 2009, als China in einem großen Sprung den Abstand zwischen dem in einer tiefen Rezession befindlichen Westen und der eigenen weiter deutlich wachsenden Wirtschaft mächtig reduzieren konnte, herrscht ein neuer Systemwettbewerb. Dieser wurde und wird durch Projekte wie das der geplanten aber gescheiterten transpazifischen Handelspartnerschaft zwischen Pazifikanrainern inklusive der USA aber ohne China, durch die chinesische Initiative der Neuen Seidenstraße oder durch offen ausgetragene Rivalitäten im Bereich der 5G-Neztwerktechnologien weiter befeuert. Die dahinter liegende Doktrin ist die des „War By Other Means“: der Verwendung von Handelspolitik zur Verfolgung machtpolitischer Interessen in einer Nullsummenlogik. Auch in Deutschland führte das gestiegene Misstrauen zu mehreren Verschärfungen der Außenwirtschaftsverordnung und zu einer neuen Debatte um technologische und wirtschaftliche Souveränität, die letztlich sowohl in den USA als auch in Europa eine Eindämmung des Einflusses politischer und wirtschaftlicher Rivalen, insbesondere Chinas, zum Ziel haben.

Im Zuge dieser Entwicklungen kam es schon im zurückliegenden Jahrzehnt zu einer Verkürzung der Wertschöpfungsketten. Steckten in 1 Euro deutscher Exporte 2011 nach Zahlen der OECD 77 Eurocent heimischer Wertschöpfung, so waren es im Jahr 2016 schon 80 Eurocent und 2019 schätzungsweise 81. In den USA stieg der Anteil heimischer Wertschöpfung auf mittlerweile 92 %; in China kletterte er von weniger als 60 % im Jahr 2000 auf mindestens 85 % im Jahr 2019.

Die Corona-Krise verschärft die geopolitischen Spannungen. Der Internationale Währungsfonds (IMF) rechnet in den alten Industriestaaten 2020 und 2021 mit einer Schrumpfung des realen Bruttoinlandsprodukts (BIP) um insgesamt 1,6 % während China in den beiden Jahren um insgesamt 10,4 % wachsen soll (IMF, 2020). Der Abstand zwischen den Bruttoinlandsprodukten der Blöcke schrumpfte mithin in zwei Jahren um 12 %. Dieser Terraingewinn Chinas wird in den USA mit großem Argwohn beobachtet; Präsident Trump suggeriert, dass das Coronavirus gerade für diesen Zweck von einem Labor in Wuhan in die Welt gesetzt worden sei.

Dazu kommt, dass die Verlangsamung des Wirtschaftswachstums in China die Erfüllung des Handelsabkommens mit den USA, das Verpflichtungen zum Kauf großer Mengen US-amerikanischer Güter vorsieht, stark erschwert, vielleicht sogar verunmöglicht. Präsident Trump hat für diesen Fall neue Strafzölle angedroht. Hält China an den geplanten Ankäufen fest, dann kommt es zu massiven Umlenkungen seiner Importe, weg von Europa und anderen Ländern, hin zu den USA. Wie auch immer, massive Spannungen sind vorprogrammiert.

Neben diesen politischen Problemen führt die Corona-Krise selbst zu einer massiven Reduktion des Welthandels. Die WTO geht gemäß einer aktuellen Studie von einer Schrumpfung des Handelsvolumens im Jahr 2020 um bis zu einem Drittel aus (WTO], 2020). Zum einen kommt es durch behördlich angeordnete Betriebsschließungen in vielen Ländern zu Unterbrechungen von Lieferketten und damit zu Produktionsausfällen bei Industriegütern, die immer noch drei Viertel des Welthandels ausmachen. Zum anderen führen fallende Einkommen zu einem Rückgang der Nachfrage nach langlebigen Wirtschaftsgütern, deren Kauf man aufschieben kann: Autos, Elektronikgeräte, Investitionsgüter. Wie schon in der letzten großen Wirtschaftskrise wird der Handel, der ja gerade diese Güter umfasst, deutlich stärker einbrechen als die globale Wirtschaftsleistung, die größtenteils von nicht handelbaren Dienstleistungen dominiert wird (Levchenko et al., 2010). Außerdem zeichnet sich ab, dass ausländische Direktinvestitionen zurückgehen werden; nach Schätzungen der Konferenz der Vereinten Nationen für Handel und Entwicklung (UNCTAD) um 30 % bis 40 %.

### Rückbau der Globalisierung?

In Deutschland wird die absehbare, deutliche Reduktion des Welthandels von vielen Beobachtern als Beleg dafür gesehen, dass die starke Orientierung des Landes auf globale Märkte und Produktionsnetzwerke die Verletzlichkeit der Wirtschaft vergrößert habe. Vielerorts wird ein vorsichtiger Rückbau der Globalisierung als Weg zu mehr Resilienz vorgeschlagen.

In der Tat ist es denkbar, dass die durch internationale Arbeitsteilung hervorgebrachte sektorale Spezialisierung die Volatilität des gesamtwirtschaftlichen Outputs erhöhen kann. Das muss aber mitnichten so sein. Wenn die Schocks nicht sektoraler, sondern länderspezifischer Natur sind, dann hat der internationale Handel eine Versicherungsfunktion, und die makroökonomische Volatilität geht bei einem Sinken der Handelskosten zurück. Neuere Literatur weist nach, dass länderspezifische Schocks quantitativ deutlich wichtiger sind als sektorale; daher dominiert die Versicherungsfunktion (Caselli et al., 2020). Was die Corona-Pandemie angeht, so ist klar, dass es sich hierbei um einen systemischen Schock handelt, der alle Länder und Wirtschaftszweige trifft. Dem internationalen Handel kommt nur insofern eine Stabilisierungsfunktion zu, als verschiedene Länder zu unterschiedlichen Zeiten und in unterschiedlicher Intensität betroffen sind. Aus diesen Argumenten folgt mitnichten, dass eine weniger ausgeprägte internationale Arbeitsteilung die nationale Bewältigung des Schocks erleichtern würde.

Naturkatastrophen bieten ein gutes Beispiel dafür, dass die Diversifizierung durch internationalen Handel stabilisierend wirkt. Ein von einem solchen Schock getroffenes Land in Autarkie ist offensichtlich in größeren Schwierigkeiten, als wenn es Zugang zu internationalen Märkten hätte, weil es lebenswichtige Güter nicht importieren kann. Exporteure, die einen Anstieg der Preise dieser Güter in der eigenen Wirtschaft verhindern wollen, sind allerdings versucht, Exportbeschränkungen zu erlassen, was die Versicherungsfunktion des Handels zerstört.

Die mikroökonomische Literatur zu katastrophenbedingten Schocks auf einzelne Lieferanten belegt, dass solche Schocks sich zwar in Produktionsnetzwerken ausbreiten und zu erheblichen Schäden bei den Kunden führen, vor allem wenn es sich um spezifische Inputs handelt, dass aber Unternehmen, die eine gut diversifizierte globale Lieferantenstruktur haben, widerstandsfähiger sind (Todo et al., 2015).

Diese Überlegungen gelten auch im Medizinbereich, der in der Corona-Krise ganz besonders im Fokus steht. Die hier zu Recht geforderte höhere Liefersicherheit wird nicht mit einer Renationalisierung der Produktion erreicht, sondern mit einer stärkeren Diversifizierung der Beschaffungssysteme und mit höherer lokaler Lagerhaltung.

Die größten Verlierer einer Verschärfung der Krise des Welthandels und der Welthandelsorganisation wären zweifellos die Schwellen- und Entwicklungsländer. Das entwicklungspolitische Modell, auf die Einbindung in globale Produktionsnetzwerke zu setzen, wäre sehr viel schwieriger zu realisieren.

### Bedeutung für die Wirtschaftspolitik

In den vergangenen Jahren ist die deutsche Außenwirtschaftsverordnung wiederholt angepasst worden. In der Corona-Krise kam es zwischenzeitlich bereits zur nächsten Verschärfung. So wurde das Gesundheitswesen in die Liste der besonders sicherheitsrelevanten Branchen, in denen sich die Bundesregierung eine Prüfung und Untersagung ausländischer Investitionen vorbehält, aufgenommen. In einer Zeit zunehmenden Misstrauens und offensichtlich opportunistisch motivierter Handelspolitik der größten Volkswirtschaften China und USA, sind diese Verschärfungen politisch verständlich. Allerdings sollten wirtschaftspolitische Eingriffe nicht auf Basis bloßer Verdachtsmomente oder des Lobbyings bestimmter gesellschaftlicher Gruppen erfolgen. Vielmehr ist es notwendig, dass sich die Politik auf Evidenz stützt und diese der Öffentlichkeit so transparent wie möglich zugänglich macht. Die Überwachung der Außenwirtschaftsverordnung könnte z. B. einer unabhängigen Behörde nach dem

Vorbild der Monopolkommission überantwortet werden. Eine solche Delegation wirtschaftspolitischer Verantwortung könnte sicherstellen, dass die Verordnung nicht populistisch missbraucht wird.

In der Corona-Krise werden Exportkontrollen aus der handelspolitischen Mottenkiste hervorgeholt. Anfang März 2020 haben Deutschland und Frankreich kurzzeitig neue Barrieren für den Export von Atemmasken und anderen medizinischen Artikeln erlassen, unter Missachtung der Regeln des europäischen Binnenmarkts. Die Nachbarländer Schweiz und Österreich protestierten vehement in Brüssel; erfolgreich. Nunmehr gelten diese Kontrollen zwar nicht mehr im Binnenmarkt, dafür aber gegenüber Drittstaaten. Mittlerweile haben mehr als 50 Länder solche Maßnahmen eingeführt. Das ist ein Problem, denn solche Restriktionen verhindern eine outputmaximierende, arbeitsteilig organisierte Produktion der knappen Artikel und verschieben den Mangel bloß, ohne ihn wirksam zu beheben.

## Deutschland sollte sich für eine Ächtung von Exportrestriktionen einsetzen

Deutschland sollte sich dafür einsetzen, dass die auf Medizinprodukte erhobenen Zölle und Handelsbarrieren eliminiert und neue Exportrestriktionen geächtet werden. Dies ist umso bedeutender, als in den zurückliegenden Wochen viele Länder Exportrestriktionen bei Lebensmitteln verhängt haben. Dies könnte in armen Ländern, die auf Nahrungsimporte angewiesen sind, zu Engpässen führen, die tödlicher als das Coronavirus selbst sind.

Die Krise bringt einen Aspekt der Globalisierung, der von der Welthandelsorganisation vollständig ausgeblendet wird, ins Zentrum der Aufmerksamkeit. Für das Funktionieren des Welthandels und insbesondere der globalen Wertschöpfungsnetzwerke ist grenzüberschreitende Mobilität von Personen erforderlich. Nun ist es aber gerade diese Form der Globalisierung, die das Coronavirus aus China nach Europa und in die USA gebracht hat. So kam es mit jenen Geschäftsreisenden, die für deutsche Unternehmen wie Webasto in der chinesischen Provinz Hubei tätig waren, nach Deutschland. Und die engen Verbindungen der norditalienischen Modeindustrie mit China könnten die frühe Verbreitung des Virus in der Lombardei verursacht haben. Auch wenn solche kausalen Zusammenhänge nur schwer nachgewiesen werden können, ist in Zukunft damit zu rechnen, dass viele Länder an ihren Grenzen stärkere gesundheitspolizeiliche Kontrollen vornehmen. Dies kann zu Verzögerungen führen, die Immigrationsvorgänge verlangsamen und das Funktionieren der globalen Wertschöpfungsketten gefährden. Der internationale Tourismus, für den im Dienstleistungsabkommen der WTO nur wenige Regeln niedergelegt sind, käme massiv unter Druck. Das wäre für Länder, die ganz besonders auf die damit verbundenen Einnahmen angewiesen sind, verheerend.

Die Weltgesundheitsorganisation schreibt zwar völkerrechtlich verbindliche Regeln vor, die dazu dienen, „die grenzüberschreitende Ausbreitung von Krankheiten zu verhüten und zu bekämpfen“, ohne „eine unnötige Beeinträchtigung des internationalen Verkehrs und Handels“ zu verursachen (Artikel 2 der Internationale Gesundheitsvorschriften, 2005). Dieses Regelwerk ist nicht mit dem zu vergleichen, was die Welthandelsorganisation WTO im Übereinkommen über die Anwendung gesundheitspolizeilicher und pflanzenschutzrechtlicher Maßnahmen (SPS) für den Güterhandel vorsieht. Dort existieren Notifikationspflichten, Transparenzvorschriften und klar definierte rechtliche Verfahren, die dafür sorgen sollen, dass die SPS-Maßnahmen verhältnismäßig sind, keine diskriminierende Wirkung haben, und eingehalten werden. Im internationalen Personenverkehr werden in Zukunft solche Regeln wichtig. Die bessere Integration gesundheitspolitischer Aspekte in das WTO-Recht, vor allem im Zusammenhang mit Dienstleistungshandel, erscheint daher von zentraler Bedeutung.

Europa sollte ein besonderes Interesse daran haben, solche Regeln zu entwickeln, und zwar nicht nur, weil der Kontinent relativ offen ist und bleiben sollte, sondern weil es auch darum gehen muss, gute Regeln für die Außengrenzen des Schengenraums zu etablieren. Dieser könnte durch die Krise nachhaltigen Schaden nehmen. Falls es den EU-Mitgliedern nicht gelingen sollte, Vertrauen zur Qualität der Kontrollen von Außengrenzen herzustellen, käme es zu einer Erosion des Schengenraums und mithin des Binnenmarkts, was erhebliche ökonomische Kosten verursachen könnte. Um dies zu verhindern, sollte die EU für den Ausbau gesundheitspolitischer Kontrollen finanzielle Mittel zur Verfügung stellen, ähnlich wie sie das für den Schutz der Außengrenzen vor illegalen Grenzübertritten im Frontex-Programm tut.

Prof. Gabriel Felbermayr, Ph.D., ist Präsident des Instituts für Weltwirtschaft (IfW) in Kiel und Professor an der Christian-Albrechts-Universität Kiel.


Literatur

Caselli F Koren M Lisicky M Tenreyro S Diversification through trade The Quarterly Journal of Economics 2020 135 1 449 502 10.1093/qje/qjz028
Internationaler Währungsfonds IMF World Economic Outlook 2020 Washington, D.C. The Great Lockdown
Levchenko, A., L. T. Lewis und L. Tesar (2010), The Collapse of International Trade during the 2008–09 Crisis: In Search of the Smoking Gun, IMF Economic Review, 58, 214–53.
Todo Y Nakajima K Matous P How do supply chain networks affect the resilience of firms to natural disasters? Evidence from the Great East Japan earthquake Journal of Regional Science 2015 55 2 209 229 10.1111/jors.12119
Welthandelsorganisation (WTO) (2020), Trade set to plunge as CO-VID-19 pandemic upends global economy, Press Release, 8. April.
